# Progression of cyclosporine A-blood levels in experimental cats receiving a high-dose treatment protocol

**DOI:** 10.3389/fvets.2024.1444586

**Published:** 2024-10-16

**Authors:** Sarah Rösch, Anna Frommeyer, Jenny Schulte Bocholt, Denis Grote-Koska, Korbinian Brand, Reinhard Mischke

**Affiliations:** ^1^Small Animal Clinic, University of Veterinary Medicine Hannover, Foundation, Hannover, Germany; ^2^Institute of Clinical Chemistry, Hannover Medical School (MHH), Hannover, Germany

**Keywords:** ciclosporin, drug monitoring, LC-MS/MS, calcineurin inhibitor, systemic immunosuppressant, oral, drug, therapeutic blood level

## Abstract

**Background:**

Cyclosporine A (CsA) is used as a steroid-sparing or alternative immunosuppressing agent in cats with various immune-mediated diseases such as immune-mediated hemolytic anemia. Daily treatment dosages of 5–20 mg/kg have been described. Interindividual variations in CsA blood levels are known to occur. To determine when steady-state conditions are reached and thus the earliest advisable time for monitoring CsA blood levels during the course of treatment, a prospective experimental study was conducted in six healthy adult Domestic Shorthair cats.

**Materials and methods:**

Cats were treated with an oral dosage of 7 mg/kg CsA q 12 h for 10 days. On days 1, 2, 3, 5, 7, and 10 after the start of CsA administration (i.e., after 1, 3, 5, 9, 13, and 19 CsA administrations), EDTA blood was collected to measure the CsA level 12 h after the CsA administration (trough values) using high-performance liquid chromatography coupled to mass spectrometry (HPLC-MS/MS).

**Results:**

Statistical analysis revealed a significant increase in mean CsA blood levels up to day 5 (2,050 ± 964.2 ng/mL [mean ± SD], 832–3,203 ng/mL [minimum–maximum]; repeated-measures ANOVA: *p* = 0.0021), while values on days 5 and 7 did not differ significantly from CsA concentrations on day 10. CsA concentrations showed markedly interindividual variability.

**Conclusion:**

Cyclosporine A blood levels reached a steady state on day 5 of high dosages of CsA q 12 h (i.e., after nine CsA administrations), indicating that this time point is suitable for monitoring blood levels in clinical patients. Results confirmed the well-known remarkable interindividual variability of CsA, indicating the need for treatment monitoring. The assessed treatment regime resulted in significantly higher mean CsA trough levels than the target range for immunosuppressive therapy (200–600 ng/mL).

## Introduction

Cyclosporine A (CsA) is a systemic immunomodulatory and immunosuppressive agent (1–3). In general, it acts on T-lymphocyte helper and inducer subpopulations by inhibiting calcineurin in T cells, thereby suppressing the production of interleukin (IL)-2 and others, such as IL-4, IL-5, IL-6, IL-8, and IL-13 (1, 2). Humoral immunity is less affected. Other anti-inflammatory effects of CsA include reduced degranulation of mast cells, eosinophils, and basophils, reduced expression of epithelial adhesion molecules, and reduced activity of antigen-presenting cells (1, 2). CsA is attributed to have antiproliferative effects. In contrast to other immunosuppressants, no toxic side effects on the bone marrow are observed (4).

Due to its immunosuppressive properties, CsA is used effectively with varying dosage recommendations in cats with various immune-mediated diseases, including skin diseases (3) and hematological diseases such as immune-mediated hemolytic anemia (5). In general, a dose of 7 mg/kg *per os* q 24 h is recommended in cats until clinical signs subside, followed by a dose reduction to an administration every 48 h (1, 6). However, in cases of immune-mediated hemolytic anemia or pure red cell aplasia, higher CsA dosages of up to 20 mg/kg *per os* q 24 h are described (1, 7). In the authors’ small animal clinic, cats with immune-mediated hemolytic anemia or pure red cell aplasia are therefore initially treated with 7 mg/kg *per os* q 12 h.

Few studies have addressed pharmacokinetics and trough levels of CsA in cats in particular, especially concerning higher dosages (1, 8, 9). However, large interindividual variations of CsA levels after administration have been described (1). To prevent high and toxic CsA levels leading to adverse effects or levels below the therapeutic range, monitoring of CsA levels after reaching steady-state conditions is recommended. However, the time required to reach a steady state in cats with high-dose twice-daily administration is not exactly known. There are variable estimates for the control of CsA levels in the literature of 1 week (7) or 2–4 weeks (1) after start of treatment, but these refer to lower dosages and/or once-daily application.

Regarding the interpretation of CsA levels, the detection method has to be considered (10). Various methods for determining CsA levels are described in the literature. Radioimmunoassay measures CsA and several of its circulating metabolites, possibly leading to higher measured CsA levels (2, 10). In contrast, high-performance liquid chromatography coupled with tandem mass spectrometry (HPLC-MS/MS) detects only the proper drug and has no cross-reactivity with its metabolites (11). In addition, the time point of sample collection is important. In contrast to peak levels determined 2 h after CsA administration according to the literature in cats (5) similar to humans (10), trough levels representing lowest CsA levels are determined within 1 h before the drug is administered (1, 9).

The main objective of this study was to determine the days of treatment at which steady-state conditions of trough levels were reached in healthy cats after oral administration of 7 mg/kg BW CsA q 12 h using HPLC-MS/MS for CsA measurement and to determine the earliest advisable time point for monitoring CsA blood levels in cats receiving this high-dose protocol. In addition, possible adverse effects should be evaluated.

## Methods

### Study design

Six healthy, experimental Domestic Shorthair cats were treated with an oral dosage of 7 mg/kg CsA q 12 h for 10 days. On 6 days: days 1, 2, 3, 5, 7, and 10 of treatment with CsA (i.e., after 1, 3, 5, 9, 13, and 19 CsA administrations), EDTA blood was collected in the evening to measure the CsA level 12 h after the CsA administration and immediately before the administration of the following dose (trough level) using HPLC-MS/MS. Possible direct reactions to the administration of CsA, reactions to venipunctures, and possible adverse reactions were recorded based on scoring systems (Tables 1–3). Therefore, daily health checks were performed including monitoring of food and water intake as well as urine and feces output. In addition, before and at the end of the experiment (on days 0 and 10), the body condition score (BCS) was evaluated according to WSAVA nutritional assessment guidelines (12), and hematological and biochemical blood tests were carried out.

**Table 1:** Score for the reaction to the administration of cyclosporine A (CsA) to Domestic Shorthair cats.

Score	Definition of reactions
0	No reaction.
1	Light short-term reduction of wellbeing (short defensive movement, vocalization, writhing movements of the head, and salivation).
2	Moderate transient reduction of wellbeing (defensive reactions such as attempt to scratch or bite; longer-lasting salivation [<15 min]).
3	Marked and persistent reduction of general wellbeing (clear defensive reactions with scratching and/or biting; longer-lasting salivation [>15 min]).

**Table 2:** Score of adverse effects of cyclosporine A (CsA) in Domestic Shorthair cats.

Score	Adverse effects
0	No adverse effects; a score of 0 corresponds to no distress.
1	Mild (e.g., mushy stools); a score of 1 corresponds to low distress.
2	Moderate (e.g., short diarrhea and single vomiting); a score of 2 corresponds to moderate distress.
3	High-grade severity (persistent diarrhea, persistent or bloody vomiting, and persistent anorexia); a score of 3 corresponds to significant distress.

**Table 3:** Score for reactions on venipuncture for blood collection in Domestic Shorthair cats.

Score	Definition of reactions
0	Without reaction.
1	Single, short defensive movement of the limb in reaction to the puncture of the cannula; short vocal expression or vocalization or gestures of discomfort.
2	Repeated and short defensive movements of the limb, restlessness.
3	Deliberate aggressive behavior of the animal (cat tries to scratch or bite); exaggerated fear behavior.

### Cats

Six experimental Domestic Shorthair cats of the Institute of Parasitology of the University of Veterinary Medicine Hannover, Germany, were included (five male castrated, one female spayed). They were only slightly accustomed to handling and treatment by veterinarians. Age was 2.5 years (*n* = 4) and 3.5 years (*n* = 2); mean body weight was 4.4 kg ± 0.7 kg (3.4–5.1 kg; minimum–maximum). For inclusion in the study, cats had to be unremarkable in clinical behavior, clinical examination, blood count, and blood chemistry (including lipase activity). Further inclusion criteria were a negative fecal parasitological examination (flotation, sedimentation, and emigration procedure according to Baermann, Institute of Parasitology, University of Veterinary Medicine Hannover) and negative test results for feline leukemia virus antigen and antibodies against feline immunodeficiency virus (SNAP FIV/FeLV tests, IDEXX, Ludwigsburg, Germany).

### Housing of the cats

Cats were housed under well-defined and standardized experimental conditions in a separate room in the Clinic for Small Animals of the University of Veterinary Medicine Hannover, Germany. Special hygienic precautions with hand and foot disinfection were taken before entering the room. All treatments were carried out according to the Cat Friendly Practice® guidelines (12). For assessment of vomiting, defecation, and food intake, single housing was necessary. Therefore, cats were housed for at minimum up to 2 h after treatment with oral CsA, as well as in the evening and overnight in single cages with resting places, litter boxes and water *ad libitum*. During the day, after the cats had defecated and fecal consistency has been evaluated, groups of up to three cats had free access to the room with toys. During free access, shared litter boxes and water were freely available. Before the cats were returned to their individual cages and before every administration of CsA, their correct identity was checked using the cats’ microchips.

Two hours before (in the morning) and after (in the evening) the oral treatment with CsA, cats were fed their usual, standardized diet according to their body weight. Water was accessible *ad libitum* during the whole time of the experiment.

### CsA administration

The required amount of CsA (microemulsion, Atopica®, 100 mg/mL CsA, Elanco, Bad Homburg vor der Höhe, Germany) was adjusted and calculated according to the determined weight at the specific time points of CsA application (in the morning and evening). Therefore, the precise dose of the drug was filled into empty capsules using a single-channel pipette (transfer pipette S, 100–1,000 μL, Brand, Wertheim, Germany). Administration in capsules (hard gelatin capsule, transparent, size 1, Wepa Apothekenbedarf, Hillscheid, Germany, source for the capsule comply with the monograph of Ph.Eur. 10.0/0016; first administration in the morning on day 1) was opted to avoid salivation observed after orally administered liquid CsA (2). In a preliminary test, it was demonstrated that—in contrast to water resulting in softening of the capsule within 10 min after filling—no palpable alteration could be observed within 2 weeks after filling the used drug formulation (Atopica®) into the capsule.

For oral administration, the prepared capsule was dipped into a commercial creamy treat (Schleckerli®, Winston, Rossmann, Burgwedel, Germany, salmon and inulin or chicken and biotin taste). As a result, the cats ingested the capsules on their own, hidden in the treats. Successful administration was monitored by a second person (JSB or AF; Supplementary Video S1). Scores for practicability and feline behavior were introduced and documented for feeding the capsules (Table 1).

### Clinical monitoring

During the trial, results of clinical examination, weight measurements (twice daily), and assessment of general behavior were recorded daily. To assess gastrointestinal tolerance and possible side effects of CsA administration (Table 2), food intake (appetite), vomiting, defecation (quantity, color), and fecal consistency (according to Purina fecal score®) were recorded and evaluated on a daily basis (Table 2). According to the approval of the experimental study, the cats could also be treated with various supportive medications in case of gastrointestinal upset. Urination was also recorded.

### Blood collection

For blood collection, cats were carefully moved in a semilateral position and snuggled and massaged by two assistants (one at the shoulder; the other one at the hip)—and distracted by a very small licking snack while the blood sample was taken from the hind leg (vena saphena medialis). Immediately before venipuncture, disinfection was performed with two skin disinfectants (Softasept N and Braunol 7.5% solution, Braun, Melsungen, Germany). An Easy-Lance disposable cannula (22Gx 1 ¼″, WDT, Garbsen, Germany) was used for the puncture. Scores for reaction on venipuncture were recorded (Table 3). For measurement of CsA blood concentrations (starting on day 1) as well as for the blood count on day 0 (before CsA administration) and on day 10 of CsA administration (after 19 CsA administrations) blood was collected into potassium EDTA sample tubes and for blood chemistry on day 0 and day 10 into lithium heparin sample tubes (Sarstedt, Nümbrecht, Germany). After venipuncture, a bandage and subsequently if tolerated by the cats a pea-sized amount of heparin gel (Hepathrombin-Gel 30000 I.E., Teofarma Deutschland, Freiburg im Breisgau, Germany) were applied on the puncture sites to prevent phlebitis.

### CsA measurements

The EDTA blood samples for CsA measurements were frozen at −80° after blood collection. They were submitted frozen on dry ice for external examination after completion of the experiment. CsA measurements were performed in the Institute of Clinical Chemistry, Hannover Medical School (MHH), Germany. The laboratory personnel were blinded. As described before (5), for analyses, blood was prepared with the MassStar automation system (Hamilton Robotics, Hamilton Germany, Graefelfing, Germany) according to IVD-CE certified kit ONEMINUTE MassTox® Immunosuppressants (Chromsystems Instruments & Chemicals, Graefelfing, Germany). Samples were analyzed using HPLC (1260 Infinity HPLC system) coupled to a tandem mass spectrometer (6460 Triple Quad LC/MS, Agilent Technologies, Palo Alto, United States). As described before (5), a 6 + 1 multiple-point calibration was performed using isotope-labeled CsA as internal standard. Four commercial quality controls were measured several times daily. Samples with CsA concentrations exceeding the linear measurement range (15–1,880 ng/mL) were diluted according to the instruction sheet. Intra-assay and inter-assay measurement precision are (coefficient of variation, *n* = 10 measurements) 1.8 and 2.6%, respectively. Accuracy was confirmed by 4 control materials and the lowest calibrator revealing results between 91 and 104% of the assigned values.

### Remaining laboratory methods

Complete blood count was measured automatically and included hematocrit, leucocyte, neutrophil, and lymphocyte counts (Advia 2120 Hematology System, Siemens Healthineers, Germany). Baseline blood chemistry profile included the following: total protein, albumin, creatinine, urea, glucose, cholesterol concentrations as well as activities of the enzymes alanine aminotransferase and lipase (based on the substrate 1,2-Di-O-lauryl-rac-glycero-3-glutaric acid 6′-methylresorufin-ester). Measurements were performed automatically with the autoanalyzer Cobas C311 using reagents, calibrator, and quality control material of the manufacturer (Roche Diagnostics, Mannheim, Germany).

### Statistics

Statistical analyses were performed using GraphPad Prism (v 9.5.1., La Jolla, United States). Data were tested for normal distribution using Shapiro-Wilk and Kolmogorov-Smirnov tests. Data of CsA levels were normally distributed and accordingly reported as mean ± standard deviation (SD). For statistical comparison of CsA levels on days 1, 2, 3, 5, 7, and 10 (i.e., after 1, 3, 5, 9, 13, and 19 CsA administrations), repeated-measures ANOVA with Geisser-Greenhouse correction with Tukey’s multiple-comparisons test was used.

Blood values recorded on days 0 and 10, were not normally distributed and reported as median and interquartile range (IQR). For these non-normally distributed blood values, the two-tailed Wilcoxon matched-pairs signed-rank test was used as experimental design for paired data. *p* values of <0.05 were considered significant.

## Results

### CsA level

**Figure 1:**
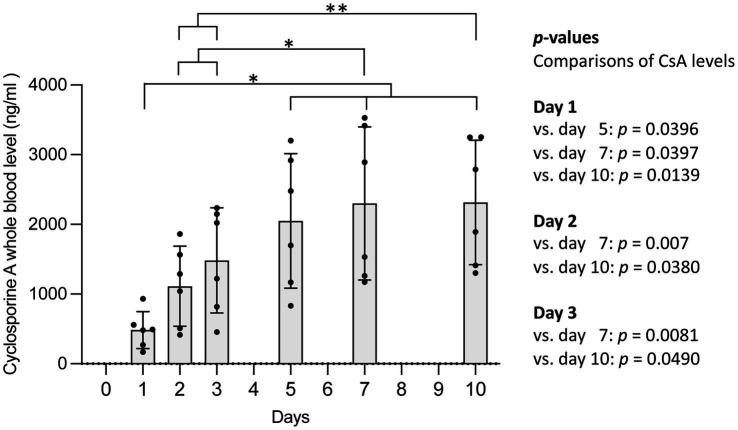
Cyclosporine A (CsA) trough levels on days 1, 2, 3, 5, 7, and 10 of treatment (i.e., after 1, 3, 5, 9, 13, and 19 CsA administrations) in EDTA blood of six healthy Domestic Shorthair cats receiving oral treatment with 7 mg/kg CsA q 12 h. Samples were taken in the evening 12 h after the first daily CsA dose before the next administration of the drug (trough levels). CsA concentrations are represented as a bar diagram (mean ± SD) and individual values as dots. The asterisks indicate statistical significance with *p ≤ 0.05, **p ≤ 0.01. vs. = versus.

The mean CsA concentration differed significantly between the six time points (days of blood sampling) with a significant increase after nine CsA administrations by day 5 (repeated-measures ANOVA, *F* = 22.21, *p* = 0.0021, Figure 1). The values on days 5, 7, and 10 (i.e., after 9, 13, and 19 CsA administrations) were not statistically significantly different. CsA concentrations showed a markedly interindividual variability (Figure 1; Supplementary Table).

### Blood examination—hematology and biochemistry

Median white blood cell counts were slightly above the reference range at the beginning and end of the study (Table 4). The absolute lymphocyte count increased significantly and was above the reference range in all cats on day 10 (Table 4). On day 10 of treatment, significant decreases in total protein and albumin concentrations were observed in all six cats compared to baseline values, even though the values (except for one cat) were within the reference range before and after treatment (Table 4). In addition, there was an increase in median blood urea concentration (Table 4).

**Table 4:** Hematological and selected biochemical parameters in six healthy Domestic Shorthair cats before (day 0) and after (day 10) administration of 7 mg/kg cyclosporine A q 12 h over a period of 10 days.

	Reference range	Unit	Median Day 0	IQR Day 0	Median Day 10	IQR Day 10	Statistical comparison (*p* values)
White blood cell count	6–12	10^3^/μL	15.7	11.8–18.2	15.8	15.1–19.2	*p* = 0.2188
Neutrophil count	3–10	10^3^/μL	9.8	6.3–10.8	7	6.4–8.1	*p* = 0.0938
Lymphocyte count	1.4–5	10^3^/μL	4.5	3.5–5.5	7.8	7.1–10.3	*p* = 0.0312*
Total protein	6–8	g/dL	7.2	6.8–8.2	6.7	6.3–7	*p* = 0.0312*
Albumin	2.6–5.6	g/dL	3.8	3.6–4	3.3	3.1–3.4	*p* = 0.0312*
Globulins	<5.5	g/dL	3.42	3.2–4.2	3.38	3.1–3.6	*p* = 0.0625
Creatinine	0–1.8	mg/dL	1.16	1.1–1.4	1.4	1.3–1.5	*p* = 0.0625
Urea	20–65	mg/dL	40	36–47	54.5	47–59	*p* = 0.0312*
Glucose	80–120	mg/dL	95	88.3–105	92.5	79.8–109.3	*p* = 0.5625
Cholesterol	70–150	mg/dL	127.5	105–136	98	79.8–116	*p* = 0.1562
Alanine aminotransferase	0–50	U/l	28	25.8–31.3	24.5	20.5–26.3	*p* = 0.0625
Lipase	0–19	U/l	19.5	13.8–22.3	14	11.8–19.3	*p* = 0.3125

Due to non-normal distribution of data, median values with interquartile ranges (IQR) are given. Statistical analysis performed by two-tailed Wilcoxon matched-pairs signed-rank test. The asterisk indicates statistical significance with *p ≤ 0.05.

### CsA administration, blood collection, toleration, and adverse effects

The cats tolerated the administration of CsA in capsules well and ate them without any fixation (scored with 0 all days; score: please see Table 1, Supplementary Video S1). No cat showed vomiting or salivation after eating the capsule. During the entire trial period, all cats showed good physical condition with vital parameters in the reference range and no clinically detectable abnormalities. All venipunctures were scored with 0–1 (score: please see Table 3). The BCS was 4/9 in all cats at the beginning and end of the study. All cats showed a good appetite and food intake during the trial. Nevertheless, one cat vomited once (without time in relation to the CsA administration). One other cat (cat 6) vomited on two occasions, which were not time-related to the CsA administration, and received a single subcutaneous injection of 1 mg/kg maropitant on day 6 and omeprazole 1 mg/kg *per os* q 24 h on the following 4 days. No further vomiting was observed in that cat. Softer feces were observed in five of six cats on the second day of CsA administration. While the consistency of the feces improved in two of five cats on the following days, the feces remained softer on most days of the experimental period in the remaining three of five cats. In one of these three cats (cat 1), a plant-based feed supplement (Diatab®; with fruit pomace, bentonite and electrolytes; Alfavet, Neumünster, Germany) was given. Therefore, adverse effects of CsA (score Table 2) were scored as 1 (3/6 cats) to medium grade 2 (3/6 cats) considering fecal quality and vomiting. In the following 6 months after the experimental trial, the cats appeared healthy in terms of general condition, behavior, food, and water intake as well as feces and urine excretion.

## Discussion

Cyclosporine A blood concentrations increased after the start of treatment with 7 mg/kg of CsA q 12 h and reached with this high-dose twice-daily administration steady-state conditions after 5 days (i.e., after 9 CsA administrations as part of a q12 h application scheme). Therefore, therapeutically relevant, blood level control of high-dose CsA treatment can be performed after this period of time. In cats with immune-mediated hematological diseases and prolonged hospitalization, this fact is important because controls of CsA levels can already be performed during hospitalization and before discharge. Previous information in the literature referring to lower dosages and/or once-daily application suggested 1 week (7) or 2–4 weeks (1) till steady-state conditions are achieved.

Astonishingly, CsA trough levels in the present study were markedly higher than the levels of the target range for immunosuppressive therapy, which are 200–600 ng/mL in “our” clinic or 400–600 ng/mL in other studies on oral CsA in cats (7, 13). The first cited range is reached in many of our feline patients suffering from immune-mediated hemolytic anemia or pure red cell aplasia and treated with 7 mg/kg q 12 h CsA (unpublished data). The first CsA blood level controls are currently performed approximately 10 days after starting therapy. The target range of 200–600 ng/mL for CsA blood levels, originally derived from human medicine, seems to be clinically effective in cats suffering from diseases cited above in “our” clinic for many years, but a retrospective assessment of these data is still pending. The markedly higher CsA levels in this present study when compared to feline patients could be due to the fact that the cats of the present study were healthy and were not receiving any additional drugs possibly influencing CsA metabolism as reported by Colombo et al. (1). Further possible influencing factors include incomplete administration compared to the optimized experimental conditions and the reduced red blood cell concentrations in anemic cats, which influences CsA blood levels as specified below. Therefore, the results of this study have to be verified in ill cats, receiving additionally other drugs. In addition, further investigations seem valuable to clarify the relevance of different factors possibly responsible for the lower CsA levels in feline patients when compared to optimized experimental conditions to optimize CsA treatment schedules in cats.

Cyclosporine A trough levels in the present study were also higher than in another study with healthy cats (2). In the cited study by Latimer et al., CsA trough values between 134 and 902 ng/mL were measured in healthy cats treated with an even higher dose of 10 mg/kg CsA q 12 h even though CsA levels were determined by radioimmunoassay (2). As already mentioned in the introduction, the detection of CsA levels by immunoassays can lead to higher CsA values by additionally detecting several of CsA circulating metabolites (5).

One possible reason for the higher CsA concentrations in our study could be the fact that cats in the cited and in other studies showed salivation (2, 5) or head shaking (2) after administration of the oral CsA solution. CsA solutions are often used for treatment in cats because they allow an exact dosing according to body weight. In contrast, this is not possible using commercially available CsA capsules (25 mg CsA per capsule) (3). To reliably administer the entire calculated dose and to avoid head shaking or vomiting after administering the CsA solution, in the present study, CsA was administered in empty capsules (hard gelatin capsules). In contrast to other studies, neither salivation nor vomiting were observed. Dipping the capsules into a commercial creamy treat resulted in easy cat-friendly administration of CsA to the cats. As a result, the cats ingested the capsules on their own, hidden in the treats. Generally, CsA is advised to be administered on an empty stomach, because its bioavailability is dependent on food (14). The advantage of a cat-friendly application of the drug clearly outweighs the theoretically minimal reduction of bioavailability by the tiny amount of the treat.

Another reason for the higher CsA values in the present study is the nature of the samples. In the study by Latimer et al. (2), CsA measurements were taken by performing radioimmunoassay on serum samples. According to Wood et al., after administration of CsA and absorption, 50% of CsA remains in red blood cells, 10–20% in leucocytes, and the remainder in the plasma, where it is mainly bound to high- and low-density lipoproteins (2). In this present study, as in other studies (10, 11), analysis was performed on EDTA blood, thereby comprising also the CsA content of blood cells (8).

One further possible reason for the higher CsA trough levels in the present study could be the formulation of CsA itself. Latimer et al. (2) used CsA in olive oil that was further diluted with peanut cooking oil, in contrast to the microemulsion Atopica® used in this present study, which is often referred to as the reference formulation (8).

The present experimental study confirmed the high interindividual variability regarding CsA trough values in cats, which has been previously described in cats (1, 2, 9) as in other species including dogs (15) and humans (16). Factors known to influence the pharmacokinetics of CsA are the absorption of CsA from the gastrointestinal tract (4), as it is only poorly absorbed after oral administration, its distribution, and hepatic metabolism (5, 17). Metabolization of the drug by enzymes such as Cytochrom-P450 in the liver is thought to be the most important factor responsible for interindividual variability (1, 18). Therefore, in sick patients – with possible gastrointestinal and hepatic alterations and additional medications – pharmacokinetics of CsA can be affected (1, 17). This can affect not only trough values but also the absorption and elimination half-life. Therefore, as already stated earlier, the earliest time interval to achieve steady-state conditions assessed in the present study should be re-evaluated in clinical patients.

One limitation of the present study and the obtained CsA levels is the small sample size of six healthy cats. This low number of experimental animals was chosen due to ethical reasons and regarding the 3R principle to reduce, refine, and replace animal experiments (19). Despite the low number of cats, the findings of the present study have a power greater than 50%, which can be extrapolated from the observed *p* values (in our study less than 0.05) with an alpha value of 0.05 (20). One more limitation is, as mentioned earlier, that the study was conducted in healthy adult cats and results may differ in diseased animals, which is why we recommend further studies in sick cats.

Trough levels were determined in the present experiment, as this is commonly used in our clinic’s patient clientele. In contrast to the evening blood sampling to determine trough levels in this experiment, appointments in the early morning before the owner administers CsA are easily practicable regarding owner communication and choice of appointment.

The specified time points (days) for the blood tests were chosen based on general pharmacological principles, suggesting that the steady state is reached after 4–6 applications, which corresponds to days 2–3 in our experiment with two CsA administrations per day (with the start of CsA application on day 1). The condition for this assumption is that the subsequent applications are below five half-lives, which was the case in this experiment, considering the result of previously conducted studies regarding the half-life of CsA [according to Schuh et al. (5); 4.6 h]. Thus, the CsA level controls were tighter at the beginning of the experiment. Due to ethical considerations evaluating the volume of blood to be withdrawn, possible skin and vein reactions with daily blood sampling, and the wellbeing of the animals, daily blood sampling was not feasible and was reduced to the specified days. In the end, however, the steady state was reached after nine CsA applications (on day 5), which is longer than usual for many other drugs. The reasons for this are speculative. Storage in fatty tissue and saturation and redistribution phenomena could possibly play a role. The duration of the trial was 10 days, as CsA levels have historically been determined in sick cats treated with CsA on this day in our clinic.

In the cats of the present study only minor post-study changes were observed in blood testing. In confirmation with Latimer et al. (2), significantly higher lymphocyte counts (above the reference range) were observed in all cats in this present study, resulting in higher leucocyte counts. Latimer et al. (2) discussed an epinephrine-induced physiological response to fixation (e.g. for blood sampling or CsA medication administration) as the cause for this, because cats can exhibit lymphocytosis during excitement. However, the authors of the present study do not consider this to be the case in the animals examined here, because firstly a synchronous increase in neutrophil granulocytes was not detected, and secondly cats were treated according to Cat Friendly Practice® without heavy fixation and with increasing adaptation to the blood sampling procedure.

In contrast to other studies with longer durations in ill cats with non-flea-induced hypersensitivity dermatitis treated with CsA, in which a slight increase in total bilirubin, a slight decrease in alanine aminotransferase activity, albumin, and alkaline phosphatase activity, and an increase in renal values (blood urea nitrogen and creatinine) were observed (1, 6, 21), all cats in this present study showed no remarkable changes in all these parameters after this present 10-day study. In agreement with other studies using higher doses of CsA in healthy cats in which hypoproteinemia was observed (1, 9), the cats in this present study showed a significant decrease in albumin and total proteins. Possibly, repeated blood sampling or gastrointestinal malabsorption of food or loss by feces are causative, especially because some cats showed gastrointestinal side effects with lower fecal consistency.

Reported adverse effects of CsA include gastrointestinal upset (vomiting, diarrhea, and anorexia), in long-term treatment gingival hyperplasia, as well as nephrotoxicity and hepatotoxicity in rats (1) or nephrotoxicity in humans (17). As a consequence of the short experimental period in the present study, gingival hypertrophy observed after 28 days of treatment in a cat in another study with CsA (2) was not observed. Soft feces was detected in most cats during the experimental period, but appetite remained good as previously described (2). Therefore, in conclusion, the side effects were comparable to other studies in which transient and self-resolving gastrointestinal discomfort was reported (9).

## Conclusion

Although the results of our study are, strictly speaking, only valid for the described protocol (solely application of CsA, special galenic formulation in empty capsules, etc.) in healthy cats, results indicate that CsA blood levels reach steady state 5 days after administration of high doses of CsA q 12 h. Therefore, results suggest that this time point is suitable for monitoring blood levels. However, this has to be confirmed in clinical patients. The used mode of application, i.e., oral CsA solution filled into empty capsules and administered with some treats, is well tolerated by cats. The results confirm the well-known, remarkable interindividual variability of CsA. The treatment regimen studied resulted in markedly higher mean CsA trough levels than the target range currently used in our clinic for immunosuppressive therapy (200–600 ng/mL) which, however, should be validated in further studies. Further studies may also clarify the main factors leading to the usually lower CsA levels in clinical patients receiving the identical CsA treatment schedule in order to possibly further optimize q 12h CsA treatment protocols in cats.

## Data Availability

The original contributions presented in the study are included in the article/Supplementary material, further inquiries can be directed to the corresponding authors.

## Supplementary material

SUPPLEMENTARY VIDEO S1 Administration of CsA-capsule in one cat. The cats tolerated the administration of CsA in capsules well. After dipping the capsules into a creamy treat, they ingested them on their own without any need for fixation. None of the cats of the present study showed vomiting or salivation after eating the capsule. As audible in the video, classical music was played throughout all treatments.

## References

[1] Colombo S Sartori R. Ciclosporin and the cat: current understanding and review of clinical use. J Feline Med Surg. (2018) 20:244–55. doi: 10.1177/1098612X17748718, PMID: 29478396 PMC10816290

[2] Latimer KS Rakich PM Purswell BJ Kircher IM. Effects of cyclosporin a administration in cats. Vet Immunol Immunopathol. (1986) 11:161–73. doi: 10.1016/0165-2427(86)90095-4 3962169

[3] Miller R Schick AE Boothe DM Lewis TP. Absorption of transdermal and oral cyclosporine in six healthy cats. J Am Anim Hosp Assoc. (2014) 50:36–41. doi: 10.5326/JAAHA-MS-5970, PMID: 24216498

[4] Gridelli B Scanlon L Pellicci R LaPointe R DeWolf A Seltman H. Cyclosporine metabolism and pharmacokinetics following intravenous and oral administration in the dog. Transplantation. (1986) 41:388–91. doi: 10.1097/00007890-198603000-00021, PMID: 3952805 PMC3000222

[5] Schuh L Kietzmann M Grote-Koska D Brand K Mischke R. Pharmacokinetics of a single orally administered therapeutic dosage of cyclosporine a in healthy cats. Res Vet Sci. (2023) 161:77–9. doi: 10.1016/j.rvsc.2023.05.015 37327691

[6] King S Favrot C Messinger L Nuttall T Steffan J Forster S. A randomized double-blinded placebo-controlled study to evaluate an effective ciclosporin dose for the treatment of feline hypersensitivity dermatitis. Vet Dermatol. (2012) 23:440. doi: 10.1111/j.1365-3164.2012.01086.x, PMID: 22882582

[7] Winzelberg Olson S Hohenhaus AE. Feline non-regenerative anemia: diagnostic and treatment recommendations. J Feline Med Surg. (2019) 21:615–31. doi: 10.1177/1098612X19856178, PMID: 31234748 PMC10814193

[8] Yang Y Kong J Liu Y Wu Q Cao Y Qiu J. Pharmacokinetics and bioequivalence of two cyclosporine oral solution formulations in cats. Front Vet Sci. (2022) 9:940472. doi: 10.3389/fvets.2022.940472 36032284 PMC9399922

[9] Roberts ES Vanlare KA Strehlau G Peyrou M Roycroft LM King S. Safety, tolerability, and pharmacokinetics of 6-month daily dosing of an oral formulation of cyclosporine (ATOPICA for cats(R)) in cats. J Vet Pharmacol Ther. (2014) 37:161–8. doi: 10.1111/jvp.12081, PMID: 24134659 PMC4282489

[10] Burckart GJ Ptachcinski RJ Venkataramanan R Iwatsuki S Esquivel C Van Thiel DH. Cyclosporine trough concentration monitoring in liver transplant patients. Transplant Proc. (1986) 18:188–93. PMID: 3538569 PMC2999908

[11] Mehl ML Kyles AE Craigmill AL Epstein S Gregory CR. Disposition of cyclosporine after intravenous and multi-dose oral administration in cats. J Vet Pharmacol Ther. (2003) 26:349–54. doi: 10.1046/j.1365-2885.2003.00496.x, PMID: 14633187

[12] Rodan I Dowgray N Carney HC Carozza E Ellis SL Heath S. 2022 AAFP/ISFM cat friendly veterinary interaction guidelines: approach and handling techniques. J Feline Med Surg. (2022) 24:1093–132. doi: 10.1177/1098612X221128760, PMID: 36259500 PMC10845437

[13] Katayama M Katayama R Kamishina H. Effects of multiple oral dosing of itraconazole on the pharmacokinetics of cyclosporine in cats. J Feline Med Surg. (2010) 12:512–4. doi: 10.1016/j.jfms.2010.02.002, PMID: 20371199 PMC10822288

[14] Fahr A. Cyclosporin clinical pharmacokinetics. Clin Pharmacokinet. (1993) 24:472–95. doi: 10.2165/00003088-199324060-00004 8513650

[15] Steffan J Strehlau G Maurer M Rohlfs A. Cyclosporin a pharmacokinetics and efficacy in the treatment of atopic dermatitis in dogs. J Vet Pharmacol Ther. (2004) 27:231–8. doi: 10.1111/j.1365-2885.2004.00587.x 15305852

[16] Savoldi S Maiorca R Chiappini R Scolari F Sandrini S. Trough cyclosporine concentration variability. Transplant Proc. (1998) 30:1642–4. doi: 10.1016/S0041-1345(98)00374-1 9723225

[17] Einecke G Mai I Diekmann F Fritsche L Neumayer HH Budde K. Cyclosporine absorption profiling and therapeutic drug monitoring using C(2) blood levels in stable renal allograft recipients. Transplant Proc. (2002) 34:1738–9. doi: 10.1016/S0041-1345(02)03003-8, PMID: 12176557

[18] Lindholm A. Factors influencing the pharmacokinetics of cyclosporine in man. Ther Drug Monit. (1991) 13:465–77. doi: 10.1097/00007691-199111000-00001, PMID: 1771643

[19] Kirschner KM. Reduce, replace, refine-animal experiments. Acta Physiol (Oxford). (2021) 233:e13726. doi: 10.1111/apha.13726, PMID: 34418296

[20] Hoenig JM Heisey DM. The abuse of power: the pervasive fallacy of power calculations for data analysis. Am Stat. (2001) 55:19–24. doi: 10.1198/000313001300339897

[21] Steffan J Roberts E Cannon A Prelaud P Forsythe P Fontaine J. Dose tapering for ciclosporin in cats with nonflea-induced hypersensitivity dermatitis. Vet Dermatol. (2013) 24:315–322.e70. doi: 10.1111/vde.12018, PMID: 23530522 PMC7169265

