# Supplementary material for: Progression of cyclosporine A-blood levels in experimental cats receiving a high-dose treatment protocol
Source: Front Vet Sci. 2024 Oct 16;11:1444586. doi: 10.3389/fvets.2024.1444586 (PMC11521867; doi:10.3389/fvets.2024.1444586)
Supplement: Supplementary file 2 [file Data_Sheet_1.pdf]

Supplementary Files

Supplementary Table

Cyclosporine A (CsA) trough levels (ng/ml) on days 1, 2, 3, 5, 7, and 10 of treatment (i.e., after 1, 3, 5, 9, 13, and 19 CsA administrations) in EDTA blood of six healthy Domestic Shorthair cats receiving oral treatment with 7 mg/kg CsA q 12 h. Samples were taken 12 h after the first daily CsA dose before the next administration of the drug (trough levels). Due to normal distribution data are shown as mean ± SD. SD = Standard deviation.

	**Cat 1** ng/mL	**Cat 2** ng/mL	**Cat 3** ng/mL	**Cat 4** ng/mL	**Cat 5** ng/mL	**Cat 6** ng/mL	**Mean ± SD** ng/mL
**Day 1**	168	483	490	271	930	558	483 ± 264
**Day 2**	415	1287	1862	508	1565	1043	1113 ± 575
**Day 3**	819	2020	2147	456	2236	1222	1483 ± 756
**Day 5**	1169	3203	2918	832	2477	1699	2050 ± 964
**Day 7**	1263	3416	3529	1173	2891	1530	2300 ± 1099
**Day 10**	1302	3253	3248	1412	2787	1891	2316 ± 893

Supplementary Video: Administration of CsA-capsule in one cat

The cats tolerated the administration of CsA in capsules well. After dipping the capsules into a creamy treat, they ingested them on their own without any need for fixation. None of the cats of the present study showed vomiting or salivation after eating the capsule. As audible in the video, classical music was played throughout all treatments.
